# Lineage-Specific Genes and Family Expansions in Dictyostelid Genomes Display Expression Bias and Evolutionary Diversification during Development

**DOI:** 10.3390/genes12101628

**Published:** 2021-10-16

**Authors:** Saara K. Luna, Frédéric J. J. Chain

**Affiliations:** 1 Department of Biological Sciences, University of Massachusetts Lowell, 1 University Ave., Lowell, MA 01854, USA; saara_luna@fas.harvard.edu; 2 Department of Molecular and Cellular Biology, Harvard University, 52 Oxford Street, Cambridge, MA 02138, USA

**Keywords:** gene duplication, duplicate gene evolution, paralogs, positive selection, *Dictyostelium*, multicellular evolution, comparative transcriptomics, expression specificity, lineage-specific expansion, developmental stage expression

## Abstract

Gene duplications generate new genes that can contribute to expression changes and the evolution of new functions. Genomes often consist of gene families that undergo expansions, some of which occur in specific lineages that reflect recent adaptive diversification. In this study, lineage-specific genes and gene family expansions were studied across five dictyostelid species to determine when and how they are expressed during multicellular development. Lineage-specific genes were found to be enriched among genes with biased expression (predominant expression in one developmental stage) in each species and at most developmental time points, suggesting independent functional innovations of new genes throughout the phylogeny. Biased duplicate genes had greater expression divergence than their orthologs and paralogs, consistent with subfunctionalization or neofunctionalization. Lineage-specific expansions in particular had biased genes with both molecular signals of positive selection and high expression, suggesting adaptive genetic and transcriptional diversification following duplication. Our results present insights into the potential contributions of lineage-specific genes and families in generating species-specific phenotypes during multicellular development in dictyostelids.

## 1. Introduction

Gene duplication is a common source of new genes [1]. Gene duplications occur frequently via mechanisms such as unequal crossing-over during homologous recombination, nonhomologous end joining during DNA repair, and retrotransposition [2,3]. These mutational events can result in variation in gene content across species, in part due to lineage-specific gene expansions [4]. Following duplication, duplicate genes can undergo divergence from one another through the accumulation of mutations; as new mutations are likely deleterious, most duplicate genes are eventually pseudogenized or lost in the span of a few million years [5]. However, in some cases mutations lead to the retention of duplicate genes; for example, if a beneficial mutation leads to a new adaptive function (neofunctionalization) or if the functions of the original single gene are lost in a complementary fashion across duplicate genes, subdividing the ancestral functions between the duplicate copies (subfunctionalization) [6]. Divergence of duplicate genes can occur at the level of the protein-coding sequence and changes in gene expression [7,8,9,10], wherein the expression behavior of a gene prior to duplication can influence its probability of retention and opportunities for subfunctionalization [11,12]. New genes, in particular those that arise de novo, are expected to emerge with low levels and narrow expression across tissues in multicellular organisms [13,14]. Biased tissue expression is also commonly observed soon after gene duplication [15,16,17,18,19], which could reflect functional specialization via neofunctionalization or subfunctionalization.

Across species, gene families can be classified as orthologs (genes in different species descended from a common ancestor) and paralogs (genes arising from duplication events within species). Recent gene duplications can result in lineage-specific genes and expansions relative to other lineages, as can differential gene loss across species. The same is true for de novo genes that arise independent of duplications [20,21,22], and therefore comparative genomic approaches are useful in identifying new genes that are restricted to a single lineage. Rapid diversification is typical for new genes [23], contributing to novel functions and to gene family expansions that shape the evolution of phenotypes [18,24,25]. For example, the diversification of opsin genes has allowed adaptation to various light environments [26], and the ability to detect and differentiate between odor molecules has occurred via the expansion, contraction, and differentiation of olfactory receptor genes [27]. The analysis of gene numbers and expression within a family gives us insights into lineage-specific expansions and subsequent functional and regulatory diversification that can lead to species-specific adaptations [24].

In this study, a genome-wide analysis of protein-coding gene family evolution and expression was conducted to identify lineage-specific genes (LSGs) and determine divergence among lineage-specific expansions (LSEs) in the dictyostelid protist group. Gene family expansions in different protist lineages are known to be involved in the adaptive development of signaling networks [28], adaptation to high salt environments [29], and adaptive evolution of social genes [30]. Dictyostelids are slime molds belonging to a large basal group of social amoebae that have a complex life cycle transitioning from a single-celled state to forming multicellular aggregates in stressful environmental conditions. Dictyostelid species for which sequenced genomes are available have diverged around 500 million years ago [30,31]. Despite deep divergence times, there remains remarkable synteny among some species [30], and the transition to multicellularity involves numerous conserved genes both in sequence and expression [32,33,34]. Here we have identified LSGs and gene families that have undergone LSEs in dictyostelids to determine whether relatively new genes differentially contribute to multicellular transitions by analyzing their expression profiles across development. Due to the long divergence times among species, only some lineage-specific genes are expected to be recently emerged genes, but nonetheless unique to one lineage compared to the four others. We combine the analysis of gene expression and molecular analysis of gene families across the phylogeny to determine the expression of lineage-specific genes across developmental stages, in particular expression bias in which a gene is predominantly expressed in a single developmental stage. This information was used to help characterize gene expansions under positive selection that might be involved in species-specific adaptations during multicellular transitions and contribute to phenotypic diversification.

## 2. Materials and Methods

### 2.1. Gene Families and Lineage-Specific Genes

Protein sequences were downloaded from Ensembl Protists [35] for all genome-predicted protein-coding genes in five dictyostelid species, herein referred to as dd: *Dictyostelium discoideum*, dp: *Dictyostelium purpureum*, df: *Dictyostelium (Cavenderia) fasciculata*, dl: *Dictyostelium (Tieghemostelium) lacteum*, and pp: *Polysphondylium (Heterostelium) pallidum* (Supplementary Table S1). The sequences were obtained from Ensembl release 45 except for *P. pallidum* (from release 44) as this species was not included in more recent releases. The orthology inference tool OrthoFinder version 2.3.3 [36] was used to predict orthology and paralogy relationships between sequences from the five species, creating gene families or “orthogroups”. Protein sequence IDs were converted into the corresponding gene sequence IDs, and the few duplicate entries resulting from isoforms of the same gene were eliminated. Genes were categorized into four groups: (1) shared singletons are genes without paralogs that have an ortholog in at least one other species, (2) shared paralogs are genes with paralogs that have an ortholog in at least one other species, (3) lineage-specific genes (LSGs) are genes with or without paralogs found in a single species (no orthologs detected), and (4) lineage-specific expansions (LSEs) are gene families of >4 paralogs in one species with at least twice as many paralogs as any other species.

### 2.2. Sequence Analysis

Nucleotide coding sequences were downloaded from Ensembl Protists in addition to the protein sequences used for determining orthogroups. Protein alignments from OrthoFinder were used as input for calculating protein distances using PROTDIST version 3.697 from the phylip package [37]. Mean pairwise distances were calculated among all paralogs within an orthogroup for each species. Nucleotide coding sequences had their codons aligned according to the protein alignments with PAL2NAL version 14.1 [38] and were used for phylogenetic reconstruction using FastTree version 2.1.8 [39] in the ete-evol tool version 3.0.0b36 [40]. Molecular evolution analysis testing for positive selection was carried out for biased genes with paralogs (LSEs and shared paralogs) using a branch-site model [41] as implemented in PAML version 4.8a [42] within the ete tool. Branches were considered to be under positive selection using FDR-corrected *p*-values < 0.05 between the null and alternative evolutionary models.

### 2.3. Transcriptional Analysis

Expression for each gene for each species was obtained from dictyExpress [43] corresponding to five distinct developmental stages: vegetative growth (0 h), aggregation (8 h), mound (12 h), early fruiting body (20 h), and late fruiting body (24 h), as described previously [32,33,34]. Gene expression was converted to TPM (transcripts per million) and compared among genes after excluding genes with TPM values less than 1 in all stages. Because gene IDs differed between Ensembl and dictyExpress for *D. purpureum* and *D. lacteum*, the genomic coordinates of genes were compared to identify corresponding gene IDs between the two databases. Expression bias was calculated using the expression specificity metric tau [44]. Genes were categorized as having biased expression in a species when their tau value was in the 95th percentile of all genes (as calculated for each species separately). Expression divergence among genes within orthogroups was calculated using Euclidean distances. Average pairwise divergences were calculated between genes and each of their orthologs and paralogs to determine divergence within orthogroups, and between each gene and its paralogs to determine divergence among duplicate genes.

### 2.4. Gene Ontology Analysis and Visualizations

Functions of genes were predicted using gene ontology (GO) terms from Ensembl Protists release 36. Gene ontology enrichment analysis was conducted with topGO version 2.42.0 [45] for each species and for each gene category (shared singletons, shared paralogs, LSGs, LSEs) and biased genes. Enriched GO terms were determined using an FDR-corrected *p*-value (<0.05) of the default weight01 algorithm in topGO. Figures were generated using ggplot2 (v3.3.5) [46] in R (4.0.3) [47].

## 3. Results

### 3.1. Gene Family Distribution and Lineage-Specific Genes

Across all five analyzed dictyostelid species (dd, dp, df, dl, and pp; see Methods), a total of 8531 orthogroups were determined (i.e., gene families with at least one ortholog or paralog; Supplementary Table S2). Orthogroups consist of 76% of all genes, meaning that almost a quarter of genes are singletons with no detected ortholog or paralog. Genes without orthologs are herein called “lineage-specific genes” (LSGs). On average, 54% of genes are shared singletons, 15% are shared paralogs, 24% are lineage-specific, and 7% are part of lineage-specific gene expansions (LSEs; Table 1). LSEs were recovered by determining whether the species contributing the most genes to each orthogroup has disproportionately high numbers of paralogs relative to the orthogroup size (see Methods; Supplementary Figure S1). Comparison of protein sequences revealed that LSEs have some members with high sequence conservation and others with high divergence; while protein divergence between the two most similar duplicate gene sequences within LSE orthogroups is significantly lower compared to shared paralogs (*p* < 2.2 × 10^−16^, Mann–Whitney), suggestive of recent duplications, LSEs have significantly higher average protein divergence among all duplicate gene members in their orthogroup compared to shared paralogs (*p* = 3.9 × 10^−6^, Mann-Whitney), suggesting high overall levels of protein diversification.

Of all the orthogroups, 64% (5426) have a gene member in each of the five species (which encompasses between 47–58% of genes in each genome’s repertoire), 52% (4452) are singletons in all species, less than 1% (67) are paralogs in all species, and 2% (165) are lineage-specific, meaning that they consist of two or more genes all from the same species. There are 123 orthogroups (2%) with at least 20 gene members across species, the largest orthogroup containing 148 genes (Supplementary Table S3). There are 29 of these large orthogroups that consist of genes in only two of the species, and ten large orthogroups with as many as 53 gene members are expansions of lineage-specific genes (Supplementary Figure S2).

### 3.2. Lineage-Specific Genes and Expansions Tend to Have Low and Narrow Expression

**Figure 1:**
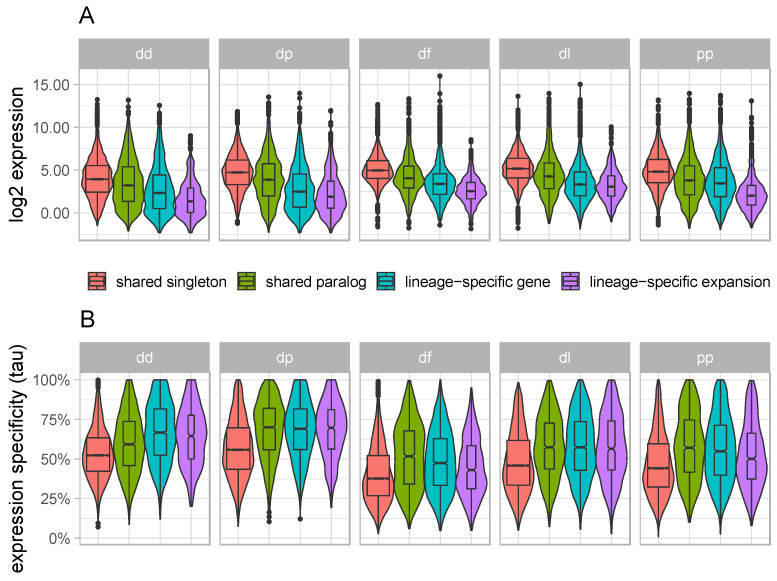
Gene expression distributions across gene categories for each of five dictyostelid species (listed in Table 1). Violin plots of (**A**) gene expression (log2 TPM) and (**B**) expression specificity (tau) for shared singletons (red), shared paralogs (green), lineage-specific genes (blue), and lineage-specific expansions (purple). dd: *Dictyostelium discoideum*; dp: *Dictyostelium purpureum*; df: *Dictyostelium fasciculata*; dl: *Dictyostelium lacteum*; pp: *Polysphondylium pallidum*.

Most genes that were filtered out with low expression (TPM < 1 in all stages) were lineage-specific (13% of LSGs and 15% of LSEs) compared to shared paralogs (9%) and shared singletons (1%). Even after excluding these genes, average gene expression was still lower among LSGs and LSEs compared to shared singletons and shared paralogs (Figure 1A). Shared singletons were almost all expressed (TPM > 1) in all five developmental stages (96–99% of shared singleton genes per genome), whereas other gene categories are on average expressed in two to four stages, with LSGs and LSEs having the narrowest average expression (Supplementary Table S4). This is reflected in the measure of expression specificity (tau), wherein LSGs and LSEs each have higher specificity than shared singleton genes (all *p* < 3.0 × 10^−8^, Mann-Whitney). Expression specificity of shared paralogs is sometimes higher (df and pp), lower (dd), or at similar levels (dp and dl) as LSGs and LSEs overall (Figure 1B). The four different gene categories are represented in consistent proportions across the five developmental stages (Supplementary Figure S3).

### 3.3. Biased Genes Are Enriched during the Early and Late Stages of Development

**Figure 2:**
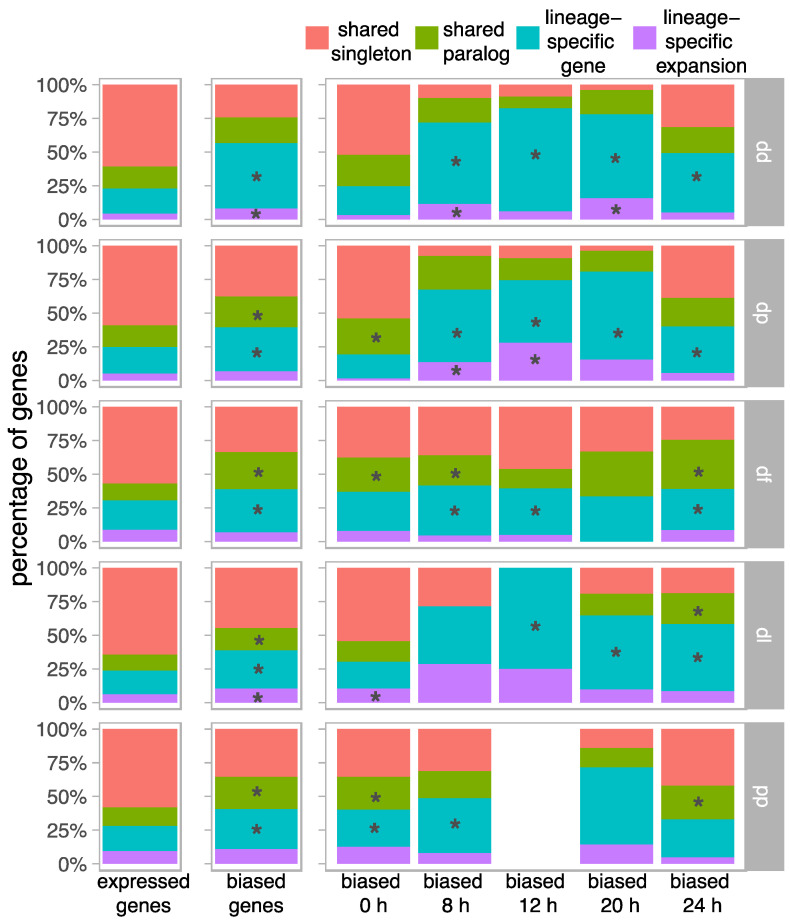
Contributions of gene categories among biased genes. Stacked bar charts showing the percentage of all expressed genes and all biased genes that belong to each of the four gene categories. Biased genes are also divided into the developmental stage in which expression bias is observed (i.e., where expression is highest): vegetative growth (0 h), aggregation (8 h), mound (12 h), early fruiting body (20 h), and late fruiting body (24 h). Asterisks (*) denote gene categories that are significantly enriched based on a chi-square test (FDR-corrected *p* < 0.05). There were no biased genes in pp at 12 h.

Genes with the most extreme expression specificity (tau in the top 5% of each species) were categorized as displaying expression bias (Supplementary Table S5). Most of the biased genes were specific to stage 0 h (43%) and 24 h (31%) compared to the other three middle stages of development (cumulative 26%). LSEs are overrepresented among biased genes in each species and almost each developmental stage, whereas shared singletons are underrepresented among biased genes (Figure 2). Half of the LSE families with biased genes (57 out of 113) contain multiple biased paralogs, compared to 21% of shared paralogs. Biased genes are proportionally more frequent in orthogroups made up of a single species (27%) than “shared orthogroups” composed of genes from multiple species (13%; *p* < 0.0001, chi-square). This results in relatively little overlap of biased orthologs across species (Supplementary Figure S4). Out of a total of 1081 shared orthogroups that contain a biased gene, only 272 (25%) have biased genes from multiple species, of which 194 (71%) are biased in the same stage. The large majority of these overlapping biased orthogroups is specific to stage 0 h (55%) or 24 h (40%) compared to the other stages (cumulative 5%).

For shared paralogs that have more than one biased gene within the same species (79 orthogroups), 79% are biased in the same stage, whereas 39% are biased in different stages (18% of orthogroups have biased genes in the same stage and in different stages). In comparison, LSEs with biased genes tend to display more expression divergence; for LSEs that have more than one member that is biased (68 orthogroups), 74% are biased in the same stage whereas 53% are biased in different stages (26% of orthogroups have biased genes in the same stage and in different stages). Among biased genes, 7% were exclusively expressed in a single stage with TPM of 0 in the four other stages; these were significantly enriched (*p* < 0.0001, chi-square) among LSEs (17% of biased genes) and LSGs (13% of biased genes), compared to shared singletons and shared paralogs (0.2% and 4% of biased genes, respectively). In addition to being narrowly expressed across developmental stages, biased genes are highly expressed compared to unbiased genes (Supplementary Figure S5).

### 3.4. Biased Duplicate Genes Display High Levels of Expression Divergence

**Figure 3:**
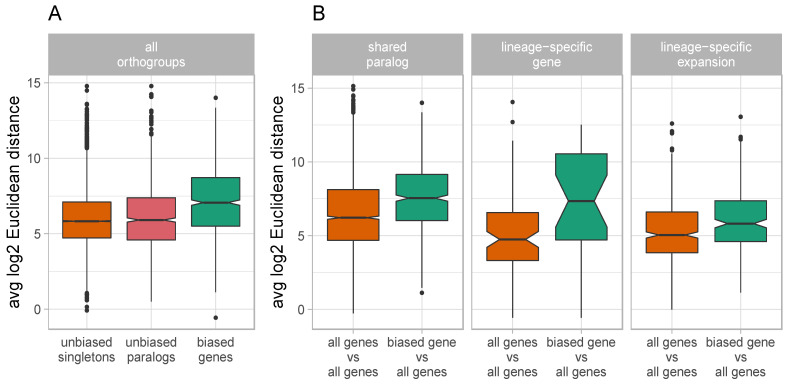
Expression divergence of duplicate genes. Boxplot of the average gene expression divergence between genes among orthogroups, as calculated using log2 Euclidean distances for (**A**) unbiased genes and biased genes, and for (**B**) paralogs across different gene categories.

Expression divergence of duplicate genes (both biased and unbiased) was on average highest among shared paralogs compared to LSGs (genes found in a single species that has up to four paralogs) and LSEs (Supplementary Figure S6). There is an overall moderate positive association between expression specificity and paralogous expression divergence (Pearson’s r = 0.18–0.40, *p* < 0.0001 for all species), meaning that duplicate genes with narrow expression across development have diverged more from their paralogs than genes with broad expression. In support of this, biased genes have greater expression divergence from their paralogs and orthologs than unbiased genes (*p* < 2.2 × 10^−16^, Mann-Whitney; Figure 3A). When calculating expression divergence among only paralogs within an orthogroup, biased genes also have significantly greater divergence than other genes do with their paralogs in all gene categories (Figure 3B), which include shared paralogs (*p* < 2.2 × 10^−16^, Mann-Whitney), LSGs (*p* = 0.013, Mann–Whitney), and LSEs (*p* = 1.9 × 10^−4^, Mann–Whitney). Biased genes had slightly lower protein distances with other gene members in their orthogroup overall (*p* = 2.1 × 10^−4^, Mann–Whitney), but the extent of this relationship differed among species (Supplementary Figure S7).

### 3.5. Gene Function and Positive Selection among Lineage-Specific Expansions

**Figure 4:**
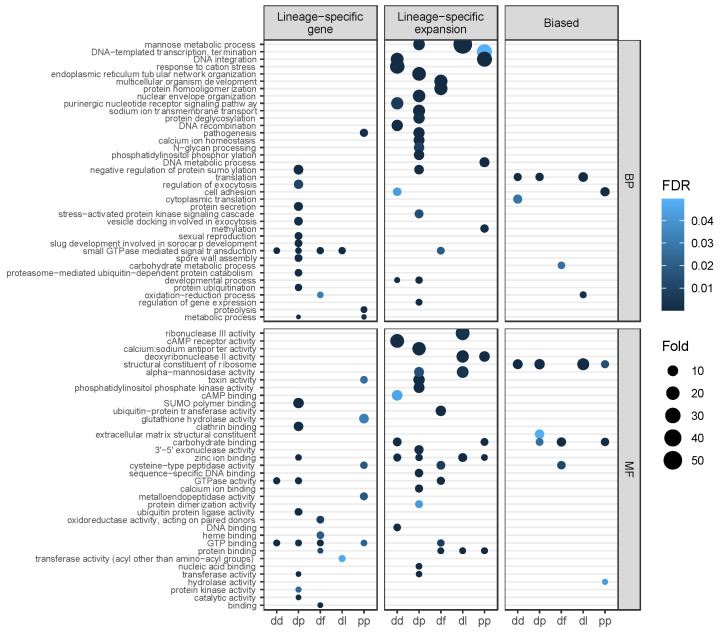
Gene ontology enrichment terms across categories. Dot plot of enriched GO terms across species for lineage-specific genes, lineage-specific expansions, and all biased genes for Biological Processes (BP) and Molecular Functions (MF). The size of the dot represents the fold difference in observed terms versus expected, and the darker the dot the lower the FDR-corrected topGO weighted *p*-value.

Gene ontology (GO) enrichment analysis was carried out to infer functions of genes among the four gene categories and for biased genes in each species. Shared singletons had a unique set of enriched GO terms, with no GO terms overlapping the set of enriched GO terms in shared paralogs, and a total of 4 (out of 69) enriched GO terms overlapping enriched GO terms in LSGs and LSEs (Supplementary Table S6). LSGs have 32 enriched GO terms across the five species, with only GTP-binding and GTPase-related functions shared among species (Figure 4). LSEs have 46 enriched GO terms, with several different functions shared across species including zinc ion binding, carbohydrate binding, alpha-mannosidase activity, DNA integration and developmental process. LSGs and LSEs share 10 enriched GO terms in common, and have several functions also enriched among biased genes (cell adhesion, oxidation-reduction process, carbohydrate binding, and cysteine-type peptidase activity). Biased genes have 10 enriched GO terms, three of which were shared in three or four species (translation, structural constituent of ribosome, and carbohydrate binding). Of the biased genes, 55% of LSEs displayed molecular signals of positive selection (FDR < 0.05) based on the branch-site model (Supplementary Table S5). These genes displayed higher expression in the biased stage than other members of their orthogroup (*p* = 0.02, Mann–Whitney), and higher average expression than biased LSEs not under positive selection (*p* = 0.0018, Mann–Whitney) but not more expression divergence (*p* = 0.4, Mann–Whitney). In contrast, 46% of biased shared paralogs were consistent with positive selection and were not significantly higher expressed (*p* = 0.065, Mann–Whitney) or diverged in expression (*p* = 0.74, Mann–Whitney).

## 4. Discussion

The core set of protein-coding genes that are shared across all five studied dictyostelid species make up about half of all genes in their genome. These are similar numbers as reported during the initial release of one of the *Dictyostelium* genomes, *D. purpureum*, in comparison with *D. discoideum* [30]. The core genes across species, especially those without paralogs, were more likely to be expressed in all five developmental stages suggesting basic shared cellular functions. But many genes are species-specific in each genome, with approximately one quarter of all genes restricted to a single species, including 165 lineage-specific orthogroups that also contain paralogs. In addition, 7% of genes are members of lineage-specific expansions in a single species, a comparable proportion to that found among *Plasmodium* species [48]. Approximately 15% of LSGs and LSEs had very low expression levels in all samples (TPM < 1) compared to 1% of shared singleton genes, plausibly because many are pseudogenes or are expressed under conditions not surveyed. While some of the identified LSGs and LSEs might be the result of gene loss in other lineages or ancient acquisition of genes with subsequent evolutionary divergence (due to large divergence times separating these species), LSEs have low protein divergence between its most similar paralogs, consistent with recent duplications.

The emergence of new genes might have allowed establishing novel phenotypes in dictyostelid lineages. Although there is a limited ability of gene ontology (GO) to inform us on the function of new genes, as older genes are typically better annotated [49], several GO terms were enriched among genes that were identified as lineage-specific. Among the most overrepresented GO terms (i.e., the highest observed:expected ratios) of LSGs were functions involved in regulating exocytosis and protein trafficking (including protein secretion, vesicle docking, clathrin binding, and ubiquitin-related functions). This might reflect functional specialization via LSGs, for example by possibly coordinating species-specific vesicle transportation and protein modification during the formation of multicellular structures, which display diverse phenotypes across dictyostelids [50]. GO term enrichment of LSGs differed among species except for GTP-related functions (e.g., GTP binding, GTPase activity, and small GTPase mediated signal transduction), which had related terms enriched in each of the species and are also enriched in LSEs. GTPases have been extensively studied in *Dictyostelium* for their importance in chemotactic signaling [51]. Their independent diversification between and within lineages might have contributed to species-specific phenotypes during development. In contrast to LSGs, LSEs share several enriched GO terms among species, suggesting evolutionary convergence of duplication and diversification of gene families with similar functions, similar to globin genes in vertebrates [52]. Shared enriched GO terms among LSEs spanned a variety of functions, including mannose metabolic process, protein binding, zinc ion binding, carbohydrate binding, deoxyribonuclease II activity, developmental process, and DNA integration. LSEs also included key functional categories related to multicellular development (e.g., multicellular organism development in *D. fasciculata*); most notably, cell adhesion, cAMP receptor activity, and cAMP binding—critical processes for chemotaxis and aggregation during development [53]—were enriched in gene expansions within *D. discoideum*, which has among the most complex multicellular development and morphology within the phylogeny [50,54]. It is possible that such gene family expansions contributed to the gain of novel phenotypes during development among different lineages.

LSGs and LSEs were found to be less broadly expressed across development than shared genes, consistent with narrow expression of new genes observed in other taxa [15,17,49,55]. The high levels of expression specificity in these gene categories contributes to their enrichment among genes with biased expression, where they display predominant expression in a single developmental stage. Half of the biased genes in LSEs also had biased paralogs, but often not biased in the same stage, indicating expression divergence after duplication. Biased genes indeed have greater expression divergence from their paralogs and orthologs than unbiased genes, suggestive of neofunctionalization or subfunctionalization. Along with the high levels of protein sequence divergence among LSEs compared to shared paralogs, our results are suggestive of functional diversification and mirror findings in plant and animal paralogs where higher tissue specificity is associated with greater expression divergence [15,19].

Less than 20% of biased genes are shared across species, indicating that stage-specific genes are mostly species-specific. GO functions enriched among biased genes were mainly driven by shared genes rather than LSGs and LSEs, suggesting conserved regulation of developmental processes at specific stages during development. These functions relate to cell adhesion, carbohydrate binding and metabolism, the structure of ribosomes and the extracellular matrix, and translation, which encompass genes important in cellular differentiation and developmental transitions in slime molds [30,56]. Lineage-specific expression bias occurs throughout development, such that each developmental stage is overrepresented with lineage-specific biased genes in at least one species: after the first growth stage, each developmental stage was enriched with lineage-specific biased genes in at least three of the five species. These findings suggest that the emergence of LSGs and LSEs contribute to several novel functions throughout multicellular development in each species, even though the functions and gene families involved are largely different across lineages. This is in line with recent evidence suggesting that novel cell types have evolved via duplications in dictyostelids [31,57]. While our methodological approach relied on the analysis of protein-coding gene sequences, there is evidence that the expression of non-coding RNA genes also plays an important role in the development and multicellularity in dictyostelids [54,58] and metazoans [59]. This includes the presence of lineage-specific RNA genes and expansions via duplications [54], which are not captured in our study and warrant further investigation as to their regulatory and functional diversification across the phylogeny.

In animals, the testis is often associated with the expression of new genes [15,17,18,60]. During dictyostelid development, biased genes were more commonly observed at the early and late stages of development, somewhat analogous to the hourglass pattern of animal expression in which middle stages of embryogenesis tends to be more conserved with low expression variation [61,62]. Interestingly, the proportion of biased genes that are LSGs is highest in the middle stages of development, potentially because they are contributing to species-specific functions in this otherwise conserved stage. If novel functions arose, many appear be related to basic cellular functions based on the enriched GO terms of biased genes, such as translation, cell adhesion, and carbohydrate binding and metabolism. Although lineage-specific genes were found to have lower average expression compared to shared genes, biased genes have higher average expression compared to unbiased genes, suggesting that biased genes that are lineage-specific are not simply narrowly expressed pseudogenes with spurious expression. In fact, we found that more than half of the biased LSE genes have molecular signals of positive selection, and these were highly expressed in their biased developmental stage even compared to orthologs, consistent with expression specialization possibly via neofunctionalization. The presence of these numerous gene family expansions unique to each dictyostelid lineage that have diverged in both sequence and expression potentially reflect the acquisition of novel species-specific expression and function throughout multicellular development.

## 5. Conclusions

Previous work has identified numerous sets of conserved genes important for developmental and multicellular processes in dictyostelids. Here we present lineage-specific genes and duplications that encompass new genes, many of which have molecular signals of positive selection and biased expression in various stages of development. Several lineage-specific gene expansions contain paralogs that have substantially diverged in protein sequence and expression from other family members, potentially mediating adaptive acquisition of novel functions and species divergence. Follow-up studies will be able to determine phenotypic impacts of these lineage-specific genes during development in dictyostelids, which are amenable organisms to knockout experiments and functional studies.

## Figures and Tables

**Table 1:** Orthologs and paralogs across five dictyostelid genomes based on orthogroup membership and size.

Species	Genes	Shared Singletons ^1^	Shared Paralogs ^2^	Lineage-Specific Genes ^3^	Lineage-Specific Expansion Genes ^4^	Lineage-Specific Expansion Families ^5^
dd: *D. discoideum*	13,243	6918	2318	3304	703	52
dp: *D. purpureum*	12,398	6782	2078	2791	747	56
df: *D. (C.) fasciculata*	12,165	6247	1483	3358	1077	78
dl: *D. (T.) lacteum*	10,224	6508	1222	1852	642	53
pp: *P. (H.) pallidum*	12,367	6255	1659	3246	1207	79

^1^ Shared singletons have orthologs in other species. ^2^ Shared paralogs have orthologs in other species but exclude lineage-specific expansions. ^3^ Lineage-specific genes include singletons and paralogs but exclude lineage-specific expansions. ^4^ Lineage-specific expansion genes have at least five paralogs in the focal species and have twice as many paralogs as other species. ^5^ Lineage-specific expansion families are the number of families (orthogroups) that contain lineage-specific expansion genes.

## Data Availability

Publicly available expression datasets were analyzed in this study. This data can be found here: https://dictyexpress.research.bcm.edu/landing/ (accessed on 25 July 2019).

## Supplementary Materials

The following are available online at https://www.mdpi.com/article/10.3390/genes12101628/s1, Figure S1: Distribution of the proportional membership of the dominant species contributor to orthogroups, Figure S2: Gene expansions of large orthogroups in one or two species, Figure S3: Expression of gene categories across development, Figure S4: Venn diagram showing the overlap of biased genes across species, Figure S5: Expression level of biased genes, Figure S6: Expression divergence among paralogs, Figure S7: Protein divergence among paralogs, Table S1: Genome assembly and annotation statistics, Table S2: Orthogroups, Table S3: Orthogroup size and genome contributions, Table S4: Average number of stages in which genes are expressed, Table S5: Biased genes, Table S6: Gene ontology terms that are enriched among gene categories in each species.
