# Supplementary material for: Lineage-Specific Genes and Family Expansions in Dictyostelid Genomes Display Expression Bias and Evolutionary Diversification during Development
Source: Genes (Basel). 2021 Oct 16;12(10):1628. doi: 10.3390/genes12101628 (PMC8535579; doi:10.3390/genes12101628)
Supplement: Supplementary file 1 [file genes-12-01628-s001.zip › Luna_GeneFamilies_SuppFigs.pdf]

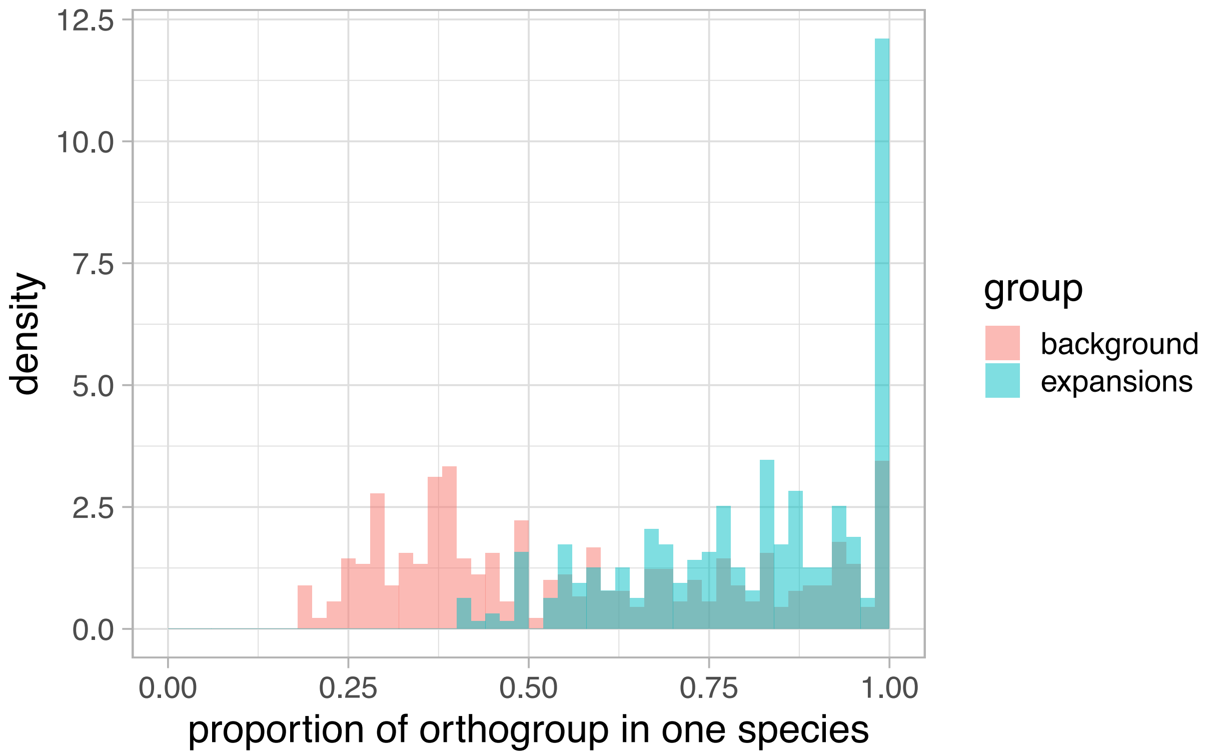

Figure S1. Distribution of the proportional membership of the dominant species contributor to orthogroups. The number (density) of orthogroups per proportion of gene members of an orthogroup that belongs to the species with the highest number of gene members. To eliminate orthogroups with equal membership across species, only orthogroups with at least 10 members are shown in red (n=450 orthogroups). Overlayed in blue is the distribution of lineage-specific gene expansions. A proportion of 0.2 would means that each of the five species contribute the same number of genes to an orthogroup (e.g., 2:2:2:2:2), whereas a proportion of 1.0 means that each a single species contributes all members to an orthogroup (e.g., 0:0:12:0:0).

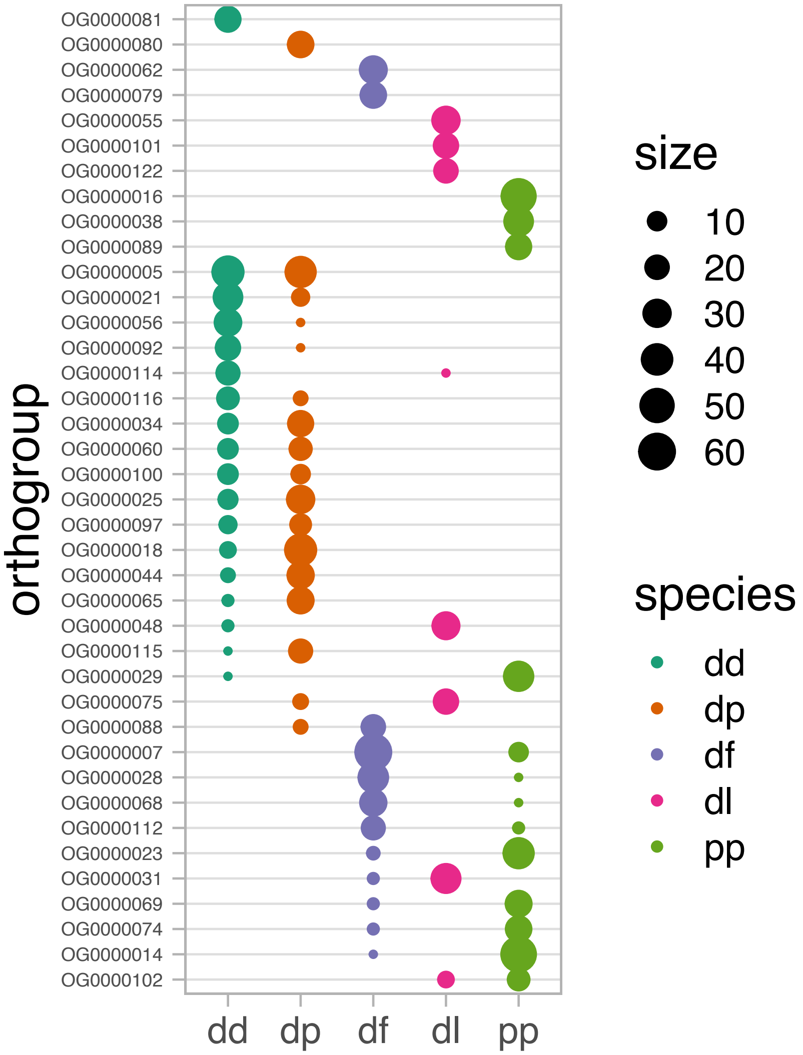

Figure S2. Gene expansions of large orthogroups in one or two species. The distribution of gene membership for orthogroups with at least 20 gene members that are from up to two of the five species.

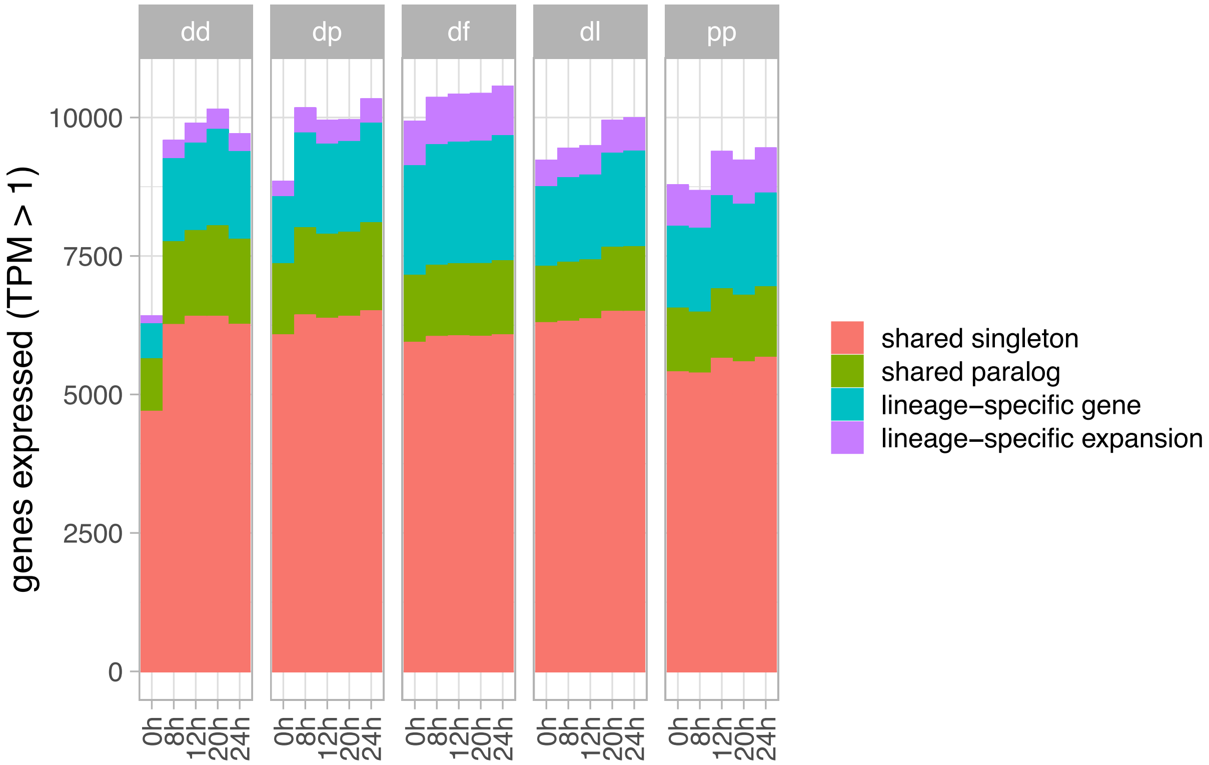

Figure S3. Expression of gene categories across development. Proportions of each of the four categories is stable across developmental stages.

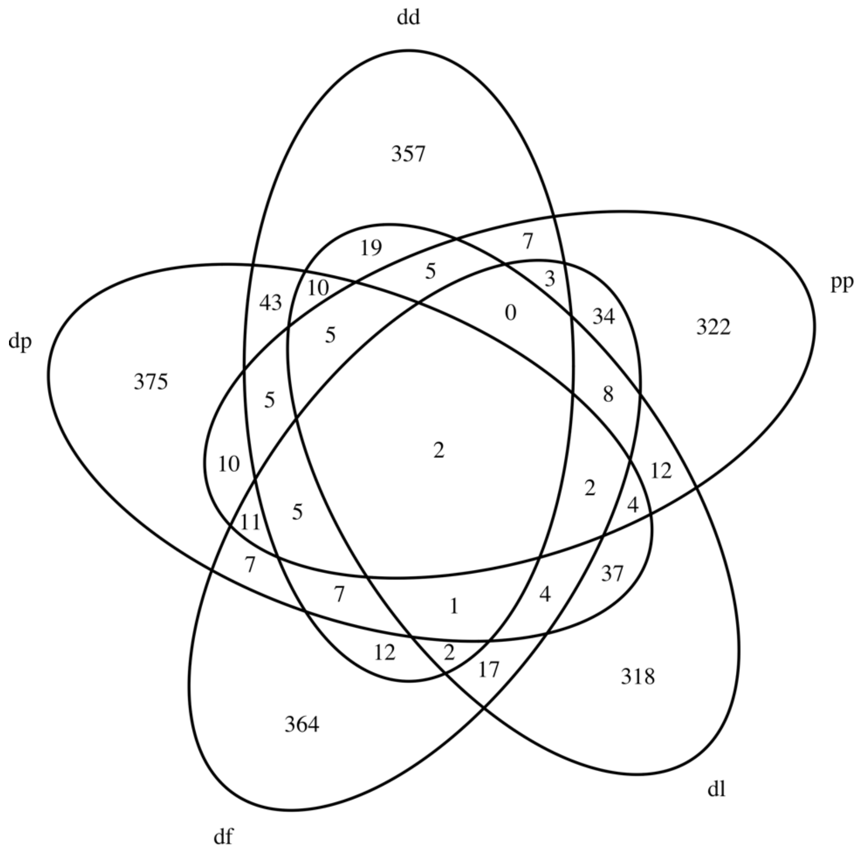

Figure S4. Venn diagram showing the overlap of biased genes across species. Most genes displaying expression bias in each species do not have orthologs (i.e., genes from the same orthogroup) that are biased in another species.

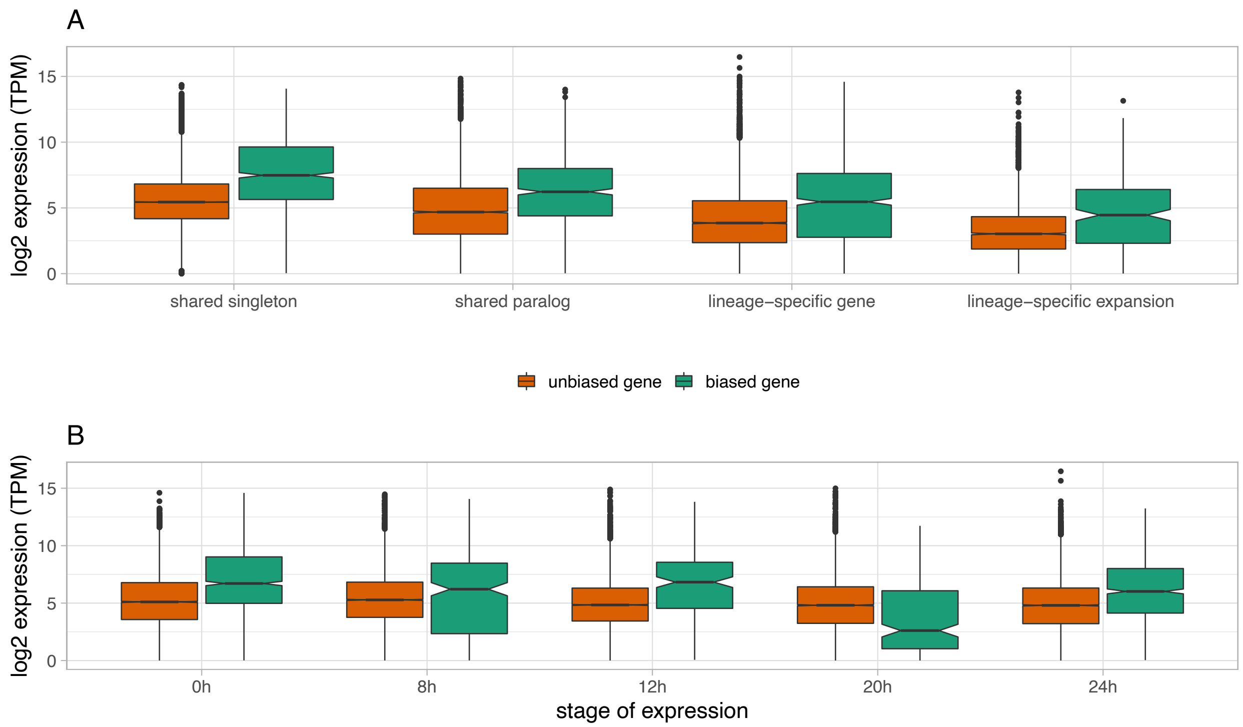

Figure S5. Expression level of biased genes. (A) Average expression of biased genes is significantly higher than unbiased genes overall and within each gene category (all with $p < 1e-9$, Mann-Whitney). (B) Average expression of biased genes is also significantly higher than unbiased genes in the biased developmental stage in four stages (0h: $p < 2.2e-16$; 8h: $p = 0.009$; 12h: $p = 7.7e-15$; 24h: $p < 2.2e-16$, Mann-Whitney), but lower in stage 20h (20h: $p = 2.1e-12$, Mann-Whitney).

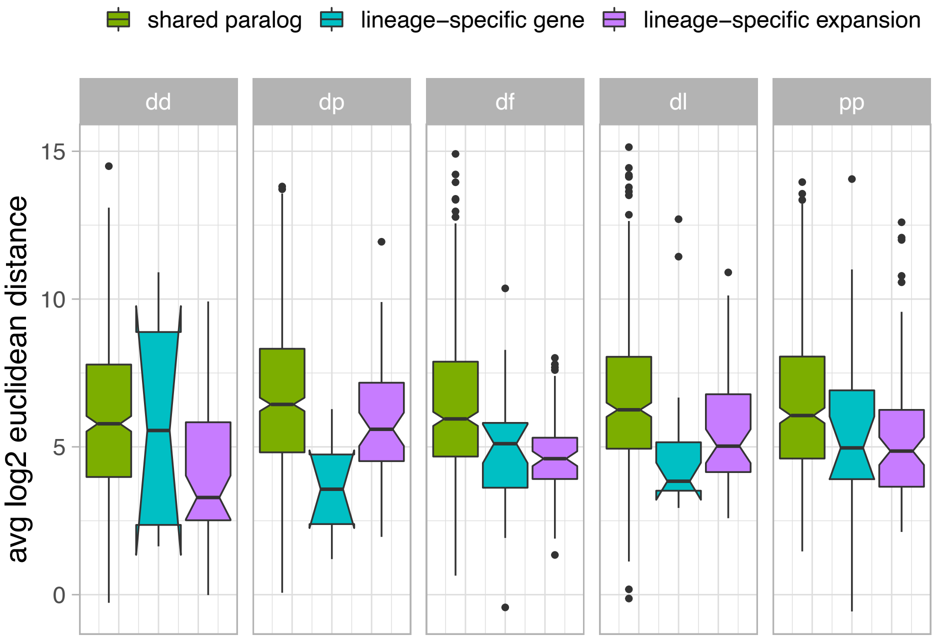

Figure S6. Expression divergence among paralogs. Divergence in expression (average log2 Euclidean distance) among duplicate genes across orthogroups, by gene category and by species.

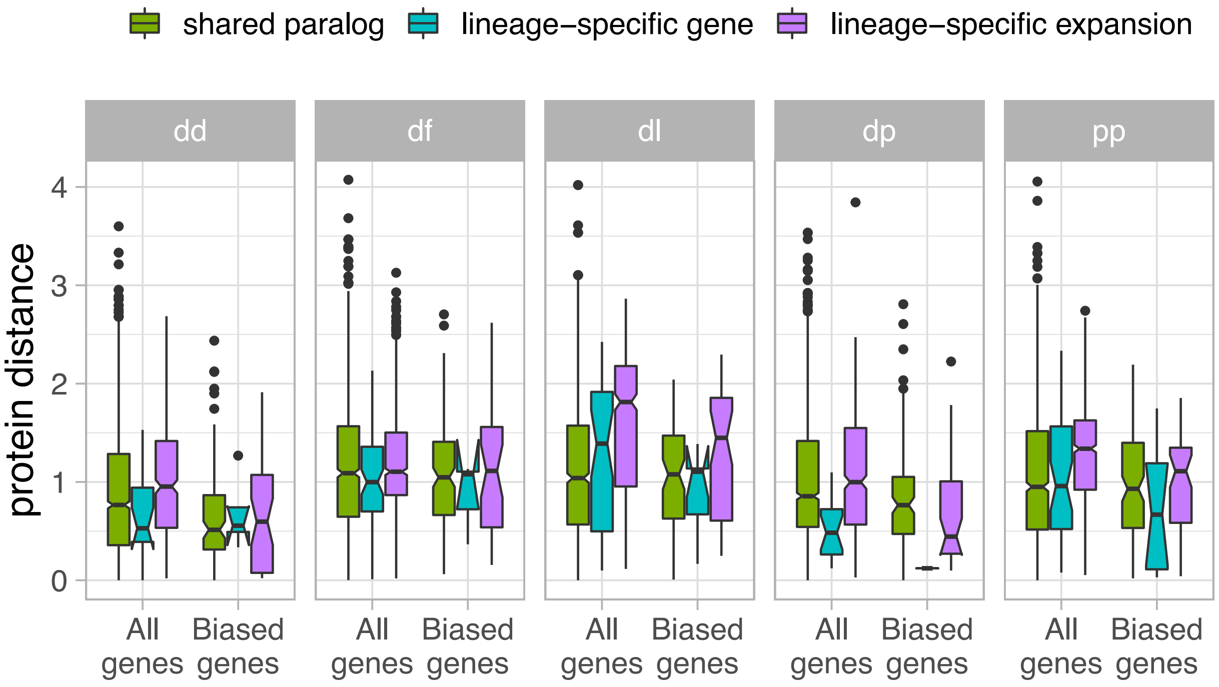

Figure S7. Protein divergence among paralogs. Divergence in protein sequences (protein distance) among duplicate genes across orthogroups, by gene category, by species, and for biased genes.
